# Anesthetic management of a huge retroperitoneal leiomyoma: a case report

**DOI:** 10.1186/s13741-023-00352-w

**Published:** 2023-11-28

**Authors:** Yue Shi, Bo Zhu, Yu Zhang, Yuguang Huang

**Affiliations:** Department of Anesthesiology, Peking Union Medical College Hospital, Chinese Academy of Medical Sciences & Peking Union Medical College, Beijing, 100730 China

**Keywords:** Retroperitoneal leiomyoma, General anesthesia, Patient blood management, Advanced hemodynamic monitoring, Case report

## Abstract

**Background:**

Retroperitoneal leiomyomas are rare, with just over 100 cases reported in the literature. Perioperative management of retroperitoneal leiomyomas can be challenging due to the large tumor size and the risk of hemorrhage.

**Case presentation:**

We report a case of a 40-year-old Han woman with a 40-cm retroperitoneal leiomyoma. General anesthesia was performed for the surgical resection. Key flow parameters like cardiac output and stroke volume variation, as shown by the Vigileo™-FloTrac™ system, enabled the anesthesiologist to implement goal-directed fluid optimization. Acute normovolemic hemodilution and cell salvage technique were used resulting in a successful en bloc tumor resection with a 6000-mL estimated blood loss. Although the patient experienced postoperative bowel obstruction, no other significant complications were observed.

**Conclusion:**

Advanced hemodynamic monitoring and modern patient blood management strategies are particularly helpful for anesthetic management of huge retroperitoneal leiomyomas.

## Background

Leiomyomas are benign tumors developed from smooth muscle cells, frequently affecting women of reproductive age in the form of pelvic lesions (Stewart et al. 2017). Traditionally, most primary retroperitoneal neoplasms are believed to be malignant, represented by leiomyosarcomas. Retroperitoneal leiomyoma is an otherwise rare condition, with just over 100 cases described in the English and Chinese literature (Poliquin et al. 2008; Fu and Zhang 2013). Primary retroperitoneal masses are often diagnosed in giant size because of their deep anatomic locations and nonspecific symptoms, which brings challenges to complete surgical resection and anesthetic management as well (Karray et al. 2018). Changes in intra-abdominal pressure during the resection are likely to cause hemodynamic instability, which must be closely monitored. An advanced discussion among the surgical team, the anesthesiology team, and blood bank should take place to cope with potential life-threatening hemorrhage. Herein, we describe a case of an en bloc resection of a large retroperitoneal leiomyoma in a woman. The application of the Vigileo™-FloTrac™ system allowed for continuous monitoring of the patient’s hemodynamic data and guiding the anesthetic management.

## Case presentation

A 40-year-old Han woman (weight 75 kg, height 160 cm) was referred to our institution with a 10-month history of increasing dull pain in the upper abdomen associated with a palpable solid mass. The patient is a married mother of three children and under regular menstruation. Her medical history was positive for a segmental pancreatectomy and cholecystectomy for pancreatic cysts 6 years ago with no remarkable comorbidities. The surgical specimen was lately considered chronic pancreatitis after consultation with a pathologist from our institution. She complained of lumbago and shortness of breath for the last 2 months, without vomiting or disturbance in bowel motility. She had no jaundice, and her urine and feces were not discolored. An evidently bulging abdomen was shown upon physical examination. The huge mass seemed to occupy the abdominal cavity between the liver and the pubic symphysis, with poor mobility and regular margins. Deep palpation of the mass caused no tenderness.

**Fig. 1:**
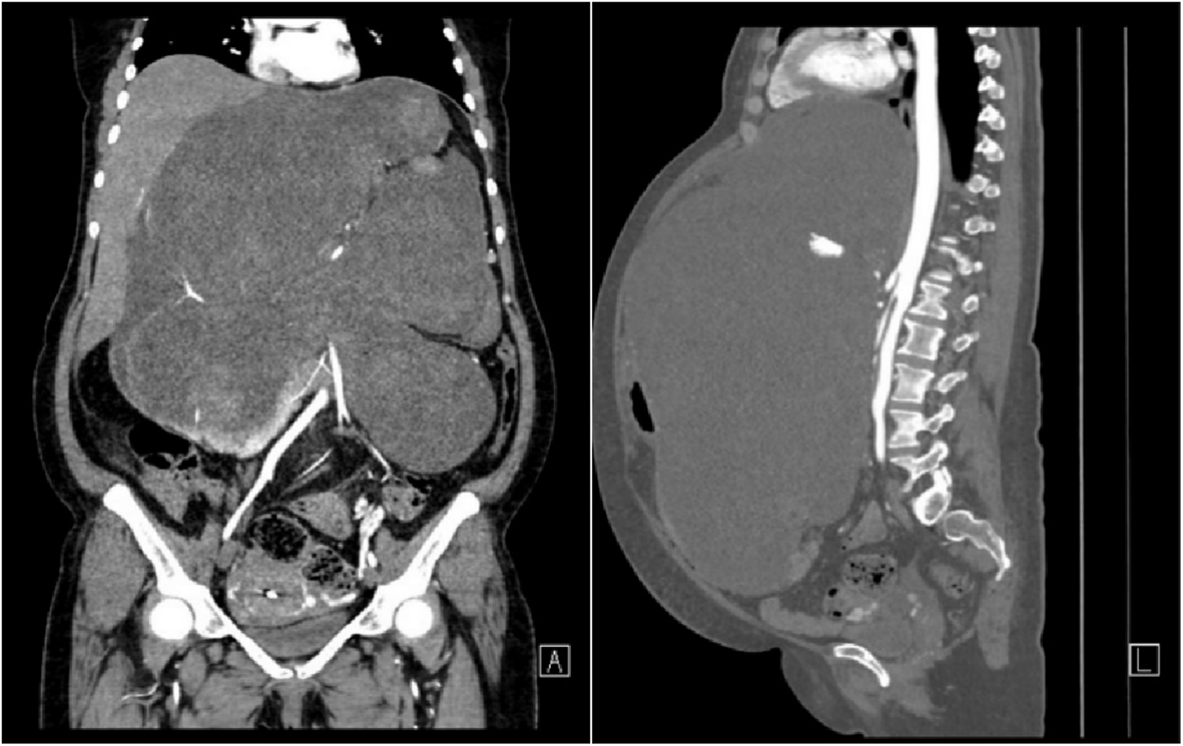
Three-dimensional reconstructed CT images of the huge retroperitoneal tumor with a mass effect on the bowel. In the arterial phase, the tumor demonstrated uneven and mild enhancement. Anterior (A). Left side (L)

The patient’s blood cell counts, renal and liver functions, and serum electrolyte levels were all within normal limits. Her hemoglobin (Hb) level was 14.0 g/dL. Her carcinoembryonic antigen level was normal, but CA-125 level was 46.8 U/mL (normal range ≤ 35.0 U/mL). She had normal resting electrocardiogram with sinus rhythm and good pulmonary function results. D-dimer test and whole leg ultrasonography were also performed to rule out venous thromboembolism (VTE). Abdominopelvic computed tomographic (CT) scan revealed a complex retroperitoneal cystic mass, which measured 32.5 × 20.1 cm at maximum cross section (Fig. 1). It was heterogeneous and mildly enhanced after contrast administration. The mass effect involved the gastrointestinal tract, the common hepatic artery, the splenic artery, and left gastric artery. A fine needle aspiration biopsy was performed, and the pathology suggested a smooth muscle tumor of unknown malignancy. The oncologists therefore considered preoperative pharmacologic treatment to be inefficient. Angiography was also performed the day before surgery and showed sparse staining of the tumor. The possible vascularization arose from left gastric artery, but the attempts at embolization failed as the opening of this artery was very sharp. A peripherally inserted central catheter (PICC) was placed from the patient’s right arm to facilitate intraoperative fluid management and postoperative nutrition support. Considering the high intra-abdominal pressure, a nasogastric tube was placed in advance to decompress and prevent recurrent vomiting.

**Fig. 2:**
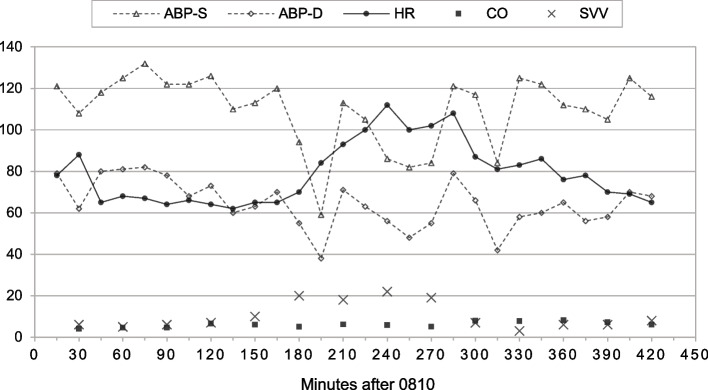
Intraoperative hemodynamic changes and FloTrac™ readings. Arterial blood pressure-systolic (ABP-S). Arterial blood pressure-diastolic (ABP-D)

When the patient arrived in the operating room, standard monitors were applied (Committee on Standards and Practice Parameters 2020). Her blood pressure (BP) was 126/101 mmHg, her heart rate (HR) was 80 bpm, and her pulse oximeter saturation was 100% while breathing room air. Baseline nasopharyngeal temperature was 36.6 °C. After a large-bore peripheral venous access was obtained, general anesthesia was induced smoothly via dexmedetomidine 0.4 μg/kg, propofol 2 mg/kg, and sufentanil 0.2 μg/kg, and subsequently, her trachea was intubated following the administration of rocuronium 0.8 mg/kg. A radial arterial catheter was inserted, and the FloTrac™ sensor (Edwards Lifesciences, Irvine, CA, USA) was attached. Key flow parameters such as cardiac output (CO) and stroke volume variation (SVV) were measured and displayed by the Vigileo™ monitor (Edwards Lifesciences, Irvine, CA, USA). Intraoperative hemodynamic changes are shown in Fig. 2.

Baseline arterial blood gas (ABG) analysis showed that initial Hb was 12.9 g/dL, and initial hematocrit was 39.7%. For acute normovolemic hemodilution (ANH), 800 mL of blood was withdrawn via the arterial catheter into standard blood transfusion bags, which was kept in the operation theater at room temperature. An internal jugular venous line was also placed after induction. During blood collection (from 0850 until 0920), approximately 1200 mL of Ringer lactate and 500 mL of hetastarch were given to maintain normovolemia. All fluids and blood were passed through fluid warmers. Her Hb level was 9.4 g/dL when ANH was completed.

Anesthesia was maintained with remifentanil 0.05 μg/kg/min, dexmedetomidine 0.4 μg/kg/h, and 2% sevoflurane in oxygen and air to keep the bispectral index between 40 and 55. After skin incision, a bolus of rocuronium 20 mg was administered every 45 min to maintain deep muscle relaxation. The patient received pressure-controlled ventilation-volume guaranteed mode, targeting an expired tidal volume of 6~7 mL/kg and an EtCO_2_ of 30~35 mmHg.

**Fig. 3:**
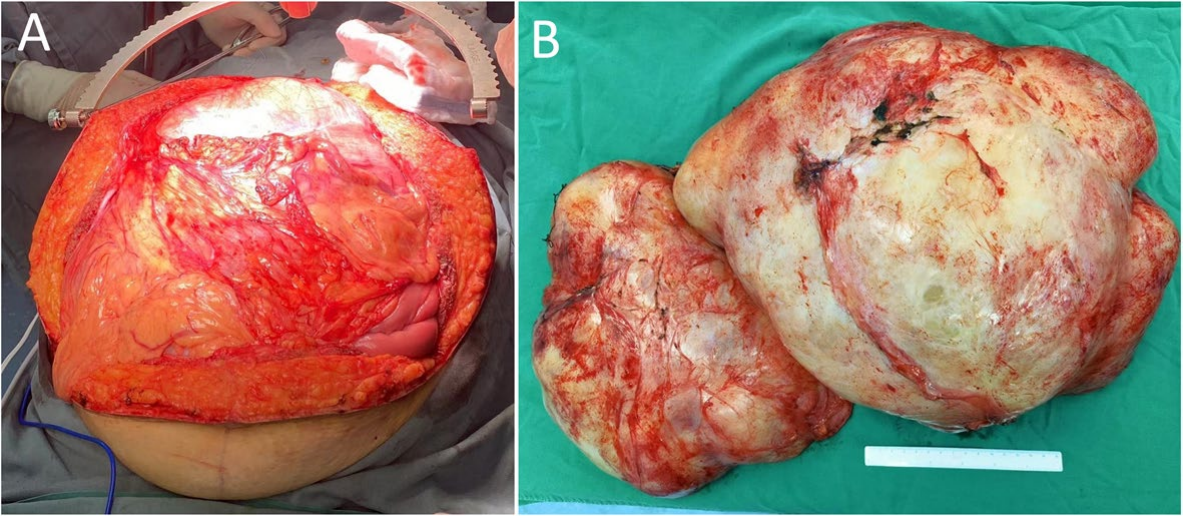
**A** A huge retroperitoneal tumor with adhesions to the peritoneum. **B** Operative specimen from en bloc tumor resection, measured 40 × 30 × 25 cm

The surgery started in the lithotomy position at 0905. A zigzag incision was made, and a huge lobulated tumor was revealed in the abdominopelvic cavity, with smooth surface and dense adhesions to the peritoneum (Fig. 3). Immediately, a suction system was used to salvage and process autologous blood in a Cell Saver® 5+ machine (Haemonetics, USA). Dissection was proceeded gradually to free the tumor from the stomach, colon, small intestine, and their mesenteries. Intra-abdominal hypertension was partially relieved by abdominal decompensation at 1010, characterized by an increase in CO along with steady HR and BP, as shown in Fig. 2.

When fully revealed, the tumor was isolated within the envelope starting from both sides and lower edge. The intention was to turn the mass upward and resect it en bloc. The main trunk of the portal vein which travelled on the surface of the tumor was torn due to the excessive weight of the mass and dense adhesions, causing severe ongoing blood loss. Clamping of the portal vein plus hemorrhagic shock resulted in a dramatic collapse in BP at 1110, and infusion of norepinephrine via central vein was initiated at the rate of 0.2 μg/kg/min. While the ruptured vessel was sutured, all ANH blood and Cell Saver units were transfused back instantly to increase the patient’s oxygen-carrying capacity. From 1110 until 1240, SVV was persistently greater than 15%, suggesting still significant hypovolemia, and thus, fluid resuscitation with crystalloids and colloids was performed via both peripheral and central veins. Meanwhile, 800 mL of plasma and prothrombin complex concentrate were given to maintain normal hemostasis. At 1310, SVV dropped below 10% again, and CO increased, accompanying with a stable BP and HR under norepinephrine 0.1 μg/kg/min, which indicated an effective fluid resuscitation. After vessel repairing completed, the lower part of the mass was isolated and excised en bloc. Subsequently, the upper part of the tumor was also carefully freed from the hepatic artery and excised intact, within the envelope.

At the end of the operation, the patient received endotracheal suctioning and manual hyperinflation. She was transferred to the surgical intensive care unit (ICU) under anesthesia at 1640. Blood loss was estimated at 6000 mL, and total urine output was 2400 mL. The Cell Saver indicated 1599 mL of packed cells returned to the patient. In addition to the ANH blood and 800 mL of plasma, the patient received eight units of allogeneic red blood cells (RBCs), 7500 mL of crystalloids, and 2500 mL of colloids during this operation. A venous blood sample taken in ICU at 1726 revealed a Hb of 12.2 g/dL. About 3 h later, she was extubated without incident. Postoperative analgesia was achieved by continuous infusion of low-dose fentanyl for the first 48 h in ICU and buprenorphine patches later. She had an ICU stay of 69 h and was discharged on postoperative day 32, which was delayed due to obstruction of the proximal jejunum.

Histological examination of the tumor demonstrated it to be a 40 × 30 × 25 cm retroperitoneal leiomyoma, with gray-red colored and a firm consistency. The tumor had degenerative changes and < 1 mitosis per 10 high-power fields, but no necrosis was seen. Immunohistochemical results showed consistency with smooth muscle tumors of uncertain malignant potential defined by Paal and Miettinen’s report (Paal and Miettinen 2001).

## Discussion

This case report describes the anesthetic management of surgical resection of a giant retroperitoneal leiomyoma. Most primary retroperitoneal masses are malignant, represented by liposarcomas and leiomyosarcomas, whereas retroperitoneal leiomyoma is benign and very rare. The incidence of leiomyomas among primary retroperitoneal tumors is estimated at 1.2%, and the largest previously documented tumor measures 37 cm at maximum diameter (Sabrine et al. 2020). In the absence of positive tumor markers or an evocative biology, diagnosis can be difficult, and percutaneous biopsy is usually necessary (Improta et al. 2020). Sometimes, surgical resection is not only for a cure or relief but also for diagnostic purpose. The deep location and large size of the tumor posed a challenge to both the surgeon and the anesthesiologist.

Management of retroperitoneal leiomyomas has been previously reported in several studies. A systematic review of 105 cases of retroperitoneal leiomyomas from 1941 to 2007 revealed a median tumor size of 12 cm, and that most were located in the pelvic retroperitoneum; there was only one case like ours where the large leiomyoma occupied both the abdominal and pelvic retroperitoneum (Poliquin et al. 2008). Karray et al. (Karray et al. 2018) reported a 22-cm retroperitoneal leiomyoma with vascularization exclusively from the renal artery. Preoperative embolization of this artery allowed shrinkage of the tumor and a one-piece resection, but the surgical blood loss and transfusions were not described in their report. Recently, Motegi et al. (Motegi et al. 2021) described an unusual case where a large retroperitoneal leiomyoma developed from the pelvic floor to the right hip was removed along with anal sphincter, with bleeding volume reaching 12,300 mL. To our knowledge, the present report for the first time provides an elaborate description of anesthesia management for giant retroperitoneal leiomyomas, including mutiple strategies for advanced monitoring and blood conservation.

Hemodynamic changes during removal of such a large retroperitoneal mass can be complex and unpredictable. We used the Vigileo™-FloTrac™ system for advanced hemodynamic monitoring, which analyzes the arterial waveform to determine CO and SVV automatically and continuously. The accuracy of this system has been tested in more than 40 studies with various results (Cannesson et al. 2007; Biais et al. 2008; Compton et al. 2008; Schlöglhofer et al. 2014; Mukkamala et al. 2021). Although overall limited agreement with reference CO determined by transpulmonary thermodilution was presented, the FloTrac™ system was still able to accurately track CO trending when preload changes or in normodynamic conditions (Meng et al. 2011; Slagt et al. 2014). Besides, SVV has proven reliable in predicting fluid responsiveness in mechanically ventilated patients, even at low tidal volumes (Alvarado Sanchez et al. 2021; Cannesson et al. 2009; Akazawa et al. 2020). In our case, changes in CO were very sensitive to changes in venous return caused by manipulating the tumor or abrupt decrease in intra-abdominal pressure. According to Fig. 2, SVV had risen to 10% from baseline level about 30 min earlier than the BP collapse at 1110 and recovered significantly when fluid resuscitation is adequate, followed by stabilization of BP and HR. Therefore, the Vigileo™-FloTrac™ system allows for effectively detecting hemodynamic changes and guiding goal-directed fluid optimization during open resection of huge retroperitoneal mass.

For major surgical procedures with a known high risk of significant blood loss (> 500 to 1000 mL), patient blood management (PBM) should be considered as a priority in perioperative management. PBM is the timely application of evidence-based surgical concepts in an effort to minimize allogeneic blood transfusion (ABT) and improve patient outcomes through three pillars, the optimization of RBC mass, reduction of blood loss and bleeding, and optimization of physiological tolerance toward anemia (Goodnough and Shander 2012; Desai et al. 2018). In this case, the giant tumor from preoperative imaging had a close anatomic relationship with surrounding organs and vital blood vessels, and its vascularization was uncertain, so there was a clear risk of high blood loss. The patient’s Hb level was measured 2 months before surgery to rule out anemia according to guidelines from the European Society of Anesthesiology and was > 13 g/dL on admission, so that optimal preoperative RBC mass was achieved (Kozek-Langenecker et al. 2017).

In terms of minimizing blood loss, we performed intraoperative cell salvage and ANH. It is suggested that use of cell salvage reduces the rate of ABT by a relative 38%, saving about 0.68 units of allogeneic RBCs per patient (Carless et al. 2010). Although there have been concerns about the negative effects of tumor cells aspirated from the surgical field, an updated meta-analysis including 34 observational studies revealed that reinfusion of salvaged autologous blood during oncology surgery was associated with reduced cancer recurrence and similar mortality compared with ABT (Frietsch et al. 2022). Preliminary safety of cell salvage was recently demonstrated in urologic malignancies, hepatocellular carcinomas, and metastatic spine tumors (Elmalky et al. 2017; Ivanics et al. 2021; Ferroni et al. 2017). Besides, leucocyte depletion filters have been shown to remove different types of tumor cells from salvaged blood, supporting more liberal use of cell salvage technique in oncology surgery (Catling et al. 2008). ANH, on the other hand, is a more cost-effective approach for intraoperative blood conservation especially in resource-constrained areas (Bansal et al. 2020). Its advantage lies in improving tissue oxygenation after surgical bleeding by whole blood re-transfusion, which also eliminates the risk of dilutional coagulopathy or thrombocytopenia. Recent studies showed that ANH is effective in reducing ABT in different surgery settings, though its efficacy of reduction of bleeding has only been demonstrated in cardiac surgery (Zhou et al. 2015; Barile et al. 2017; Stammers et al. 2017; Li et al. 2020).

The third pillar of PBM emphasizes elevating the patient’s physiological tolerance to anemia. In addition to evidenced-based transfusion strategies, cardiac output, ventilation, and oxygenation should be optimized continuously during the surgery. We used lung protective ventilation settings to maintain the oxygenation index > 300 despite the decline in Hb level. Hemodynamics were manipulated with the use of phenylephrine and norepinephrine to maintain organ perfusion. Adequate analgesia was ensured both intraoperatively and postoperatively to decrease consumption of oxygen.

The risk of perioperative VTE should be assessed for major abdominal-pelvic surgeries, and thromboprophylaxis should be individualized based on patient stratification. Malignancies are one of the major patient-related VTE risk factors (Lim et al. 2018). For our case, despite preoperative Wells score indicating a low risk of deep venous thrombosis, a D-dimer test and lower extremity ultrasound were still performed as a routine of our center for major surgeries. We used the modified Caprini risk assessment model for procedure-related VTE risk, resulting in high risk with a Caprini score of 6 (Gould et al. 2012). Given the high coexisting risk of bleeding, mechanical methods rather than no thromboprophylaxis are more recommended. We applied graduated compression stockings in the operating room and continued with venous foot pump in the ward. Ambulating after surgery was permitted once no risk of active bleeding was considered. Vena cava filters do not appear to be a routine practice for VTE prevention in surgical patients (Birkmeyer et al. 2010).

The anesthetic management of this case has several limitations. Firstly, preoperative angiography was performed, but the attempt at tumor artery embolization failed. Previous reports of huge retroperitoneal tumors indicated that arterial embolization is a safe and useful technique mainly to reduce intraoperative blood loss and to slightly reduce the tumor size (Karray et al. 2018; Burke et al. 2014). Analgesia should be strengthened after embolization, as pain is usually intense. Secondly, viscoelastic point-of-care tests, recommended for guiding the perioperative management of coagulopathy, were unavailable for this case; instead, we empirically administered platelets, fibrinogen concentrates, and prothrombin complex concentrates during the surgery.

## Conclusion

The large size and a complex anatomy of this retroperitoneal mass presented an unusual challenge for anesthetic management. Sophisticated hemodynamic monitoring through Vigileo™-FloTrac™ system and modern PBM strategies were crucial in meeting this challenge successfully.

## Data Availability

All data generated or analyzed during this study are included in this published article.
